# Stomatitis Healing via Hydrogels Comprising Proline, Carboxyvinyl Polymer, and Water

**DOI:** 10.3390/gels11020108

**Published:** 2025-02-03

**Authors:** Raichi Hanaki, Koji Harada, Yoshihiro Sasaki, Michiaki Matsumoto, Yoshiro Tahara

**Affiliations:** 1 Department of Chemical Engineering and Materials Science, Doshisha University, 1-3 Tatara-Miyakodani, Kyotanabe City 610-0321, Kyoto, Japanmmatsumo@mail.doshisha.ac.jp (M.M.); 2 Department of Nursing, Faculty of Health Sciences, Hiroshima Cosmopolitan University, 5-13-18 Ujinanishi, Minami-ku, Hiroshima City 734-0014, Hiroshima, Japan; 3 Department of Oral and Maxillofacial Surgery, Graduate School of Medicine, Yamaguchi University, 1-1-1 Minami-Kogushi, Ube City 755-8505, Yamaguchi, Japan; 4 Department of Polymer Chemistry, Graduate School of Engineering, Kyoto University, Katsura, Nishikyo-ku, Kyoto 615-8510, Kyoto, Japan; sasaki.yoshihiro.8s@kyoto-u.ac.jp

**Keywords:** stomatitis healing, hydrogel, proline, carboxyvinyl polymer, 5-fluorouracil

## Abstract

Chemotherapy using anticancer agents and radiotherapy of cancers frequently induce the development of stomatitis as a side effect. In the present study, hydrogels for effective stomatitis healing under anticancer drug administration were developed using three components, namely proline, carboxyvinyl polymer, and water (denoted proline gels). Characterization including tilting, Fourier transform infrared spectra, and viscoelasticity measurements indicated that proline gels with proline concentrations over 300 μmol/g could retain water on the tongue of mice. The degradation and release behavior of proline gels in serological environments were evaluated, revealing that proline gels were degraded by serological salt concentrations, and the cumulative amount of proline released from proline gels depended on the concentration of proline in the gel. Proline gels were applied to the stomatitis area on the tongue of mice under anticancer drug administration, with subsequent reduction in the stomatitis area and regeneration of the mucosal epithelium layer, demonstrating effective stomatitis healing by proline gels with proline concentrations over 500 μmol/g. Other control samples including the carboxyvinyl polymer or proline alone did not reduce the stomatitis area in model mice. These results suggested that the proline gel is promising for the mucosa regeneration of anticancer drug-induced stomatitis.

## 1. Introduction

Cancer has been either the first or second leading cause of death worldwide, with the incidence of cancer expected to increase by 47% over the period from 2020 to 2040 [1]. Cancer treatment includes surgery, radiotherapy, and chemotherapy using anticancer agents. Chemotherapy is important because anticancer drugs can access cancer cells spreading through the body via the circulatory system. However, the side effects of anticancer drugs are serious. For example, 5-fluorouracil (5-FU) is a widely used anticancer drug [2] that is associated with side effects such as stomatitis, diarrhea, and nausea. Among these side effects, stomatitis has a high probability of occurring, delaying cancer treatment [3,4]. Intensive chemotherapy reduces the size of the submandibular gland, with oral stomatitis emerging as a non-negligible side effect. Additionally, radiotherapy of head and neck cancers delivers radiation to or near the salivary glands, which decreases the secretion of saliva, lowering the self-cleaning ability of the oral cavity and promoting the development of oral stomatitis [5,6]. Oral stomatitis is a particularly severe problem in cancer chemotherapy because it promotes bacterial and viral infections under the weakened immunity of the patient. The pain from oral stomatitis makes speaking and swallowing difficult, which prevents the patient from ingesting water and food through the mouth, leading to dehydration and poor nutrition. In serious cases, reducing the dose of the anticancer drug can be an option, although it is not a fundamental solution. Therefore, developing effective treatments for oral stomatitis is important to improve patient quality of life during cancer therapy.

Hydrogels are commonly used as biomaterials for the treatment of wounds on the skin and mucosa. Synthetic polymers (e.g., polyvinylalcohol, polyethylene glycol, and polyglycolic acid), natural polymers (e.g., chitosan, hyaluronic acid, and gelatin), and mixtures of synthetic and natural polymers have been shown to have high therapeutic effects [7,8]. Recently, hydrogels with smart and multi-functional properties, such as polymers modified with antibacterial materials [9], photosensitizers [10,11], and thermosensitizers [12] have been reported for wound healing. However, these intelligent materials are not cost-effective for the clinical treatment of stomatitis, which is not life-threatening, although it decreases the patient’s quality of life.

In previous studies, we showed that an amino acid-rich elemental diet (Elental^®^) is effective for treating not only mucostitis induced by radiotherapy and chemotherapy in humans [13] but also skin wounds and dermatitis in mouse models [14,15,16]. However, it is difficult to keep aqueous solutions of the elemental diet in targeted areas of mucositis for a long time because, in its liquid state, the elemental diet is highly fluid. Hydrogels encapsulating these effective and inexpensive agents are promising for the practical treatment of stomatitis.

In the present study, we investigated the therapeutic effects of hydrogels consisting of only three components: proline, carboxyvinyl polymer, and water (denoted proline gels). Proline, an element of Elental^®^, exhibits high biocompatibility [17] and high solubility in water [18], which is useful in the preparation of novel hydrogels. Carboxyvinyl polymer is a branch-type cross-linked polymer based on polyacrylic acid. Although carboxyvinyl polymer also exhibits biocompatibility, its solubility in water is low. Therefore, adding carboxyvinyl polymer to water produces a viscous suspension, and adding basic molecules to this aqueous solution, which increases its pH to increase the water solubility of the polymer, produces a gel [19]. Owing to its amine and carboxylic acid moieties, proline can function as a buffer to produce hydrogels based on carboxyvinyl polymer (proline gels), and proline gels applied topically can hydrate the environments of mucositis. First, a series of proline gels with different proline concentrations was prepared and their properties, namely pH, viscosity, and rheological behavior, were characterized. Subsequently, the release of proline was investigated in vitro, and the therapeutic effect of proline gels was evaluated in vivo using mouse models of tongue stomatitis. Although there are numerous reports on the healing of whole-wound skin models, there are few reports on the treatment of mouth stomatitis models [20]. Therefore, this study is expected to provide important information for the development of novel treatments of stomatitis.

## 2. Results and Discussion

### 2.1. Characterization of Proline Gels

**Figure 1:**
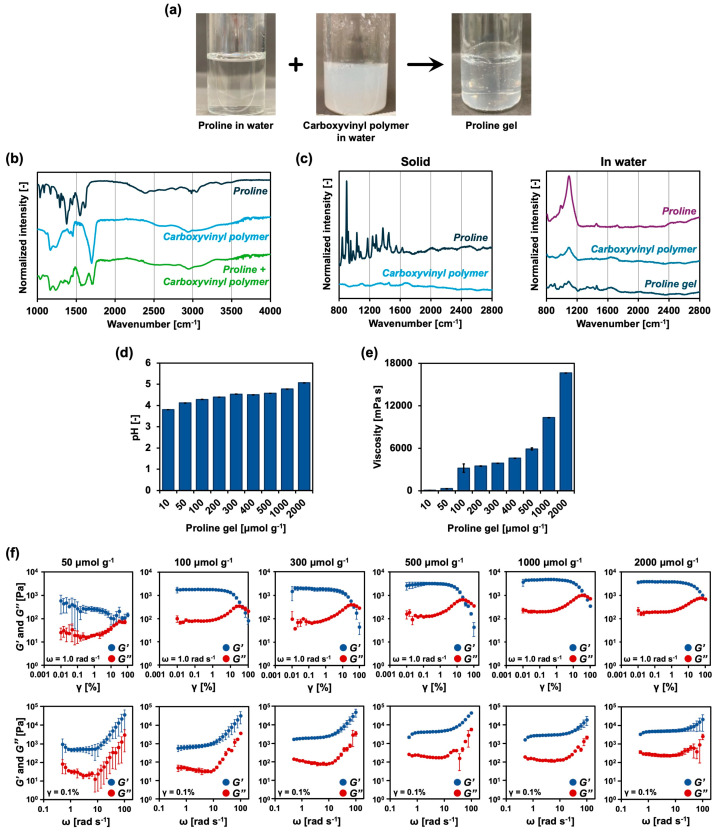
Characterization of proline gels. (**a**) Photographs of proline gels prepared by mixing aqueous solutions of proline and carboxyvinyl polymer. (**b**) FTIR spectra of proline, carboxyvinyl polymer, and the mixture of proline and carboxyvinyl polymer in the solid state. (**c**) Raman spectra of proline and carboxyvinyl polymer in the solid state and in water, and the proline gel. Effect of proline concentration on (**d**) pH, (**e**) viscosity, and (**f**) storage (*G′*)/loss (*G″*) moduli of proline gels (*n* = 3). Moduli were determined from sweeps (*n* = 3) of amplitude strain (ω = 1.0 rad s^−1^) and frequency (γ = 0.1%).

All proline gels were prepared using carboxyvinyl polymer in water at a final concentration of 0.50 wt% and proline in water at various concentrations (Figure 1a). The sol/gel states of aqueous mixtures of 0.50 wt% carboxyvinyl polymer and various concentrations of proline were evaluated using the tilting method (Table 1), indicating that proline gels could be prepared using proline in water at concentrations of 300 μmol g^−1^ to 2500 μmol g^−1^. This wide range of proline concentrations for the preparation of proline gels arises from the high solubility of proline in water [18]. To determine the qualitative strength of the proline gels, the weight at which the proline gels start to deform was measured on a contact area of 4.2 cm^2^. Both 500 and 2000 μmol g^−1^ proline gels were not deformed at 25 g per 4.2 cm^2^, while 500 μmol g^−1^ proline gel was deformed at 30 g per 4.2 cm^2^ and 2000 μmol g^−1^ proline gel was not. The strength of proline gels depends on the concentration of proline.

To clarify the gelation mechanism of proline gels, Fourier transform infrared (FTIR) spectra were acquired (Figure 1b). The peak corresponding to the symmetric stretching vibration of the amine group [21] of proline at approximately 3400 cm^−1^ was absent from the FTIR spectrum of the proline gel. In addition, the ratio of the small peak at approximately 1200 cm^−1^ to the large peak at approximately 1700 cm^−1^ [22], corresponding to the C-O-C and -C=O bonds of the carboxyvinyl polymer, was different between the spectra of carboxyvinyl polymer and proline gel. These results suggest that the interaction between the amine group of proline and the carbonyl group of carboxyvinyl polymer is important for gelation. The peak corresponding to the ionized carbonyl group at approximately 1600 cm^−1^ [23] was present in the spectrum of proline gel but absent from the spectrum of carboxyvinyl polymer, indicating that the carboxy group of carboxyvinyl polymer was liberated to form carboxy ions. These results suggest that the electrostatic interaction between the amine group of proline and the carboxy ions of carboxyvinyl polymer increases the solubility of the polymer in water, resulting in gelation of the polymer by swelling. Subsequently, Raman spectra of the proline gel, proline and carboxyvinyl polymers in solid state and aqueous solution were measured (Figure 1c). In aqueous solution, a peak of 1100 cm^−1^ was detected in all samples, and a peak of 1650 cm^−1^ was detected in both carboxyvinyl polymers and the proline gel. These results suggested that proline and carboxyvinyl polymers exist in the proline gel.

pH-induced protonation affects gelation in carboxyvinyl polymer-based systems [24]. Therefore, the pH was measured, showing that the pH slightly increased as the concentration of proline in the gel increased (Figure. 1d). However, the fluidity of the 10 μmol g^−1^ proline gel was different from that of the 1000 μmol g^−1^ proline gel, suggesting that the pH change was not related to the gelation of proline gels. Moreover, the viscosity of the proline gels increased as the proline concentration increased (Figure 1e). However, the viscosity did not change dramatically when the proline concentration was approximately 300 μmol g^−1^, which was the gelation point determined using the tilting method. The viscoelasticity of the gels was characterized by sweeping the amplitude strain (γ) and adjusting the frequency (ω) to 1.0 rad s^−1^ (Figure 1f). The observed storage (*G′*) and loss (*G″*) moduli of the proline gels were typical of those of carboxyvinyl polymer-based hydrogels [19]. Both *G′* and *G″* were unchanged when the amplitude strain was less than 1%, although *G′* decreased while *G″* increased when the amplitude strain was greater than 1%, suggesting that the polymer network of the proline gels was disrupted, and the proline gels behaved as liquids or high-viscosity materials under high shear stress. These tendencies were also observed in the frequency sweeps, with the amplitude strain adjusted to 0.1%. The difference between *G′* and *G″* in the low frequency range (ω < 10 rad s^−1^) was larger than that in the high frequency range (ω > 10 rad s^−1^). Interestingly, although both 50 and 100 μmol g^−1^ proline gels exhibited high fluidity and were classified as sols in the tilting experiment (Table 1), the difference between their *G′* and *G″* was not small, and their *G′* and *G″* increased as the proline concentration increased. These results suggest that a proline concentration of approximately 100 μmol g^−1^ is enough to form at least micro-scale partially integrated networks of proline gels, and at high proline concentrations, these gel domains are physically integrated to increase the overall viscosity of macro-scale proline gels.

As shown in Figure 1, the viscosity of the proline gel increased as the proline concentration increased, suggesting that proline gels with proline concentrations over 300 μmol g^−1^ can retain water to hydrate the tongues of mice (see Section 2.3). Therefore, the following experiments mainly used proline gels with proline concentrations from 300 to 2000 μmol g^−1^. From the overall results in Figure 1, the proline gels show a similar property to typical hydrogels, in which carboxyvinyl polymers are physically crosslinked. When dissolved in water, carboxyvinyl polymers are hydrated and the hydrogen ions begin to dissociate from the carboxy groups. The carboxyvinyl polymers form a coiled state in the resulting aqueous solution containing 0.50 wt% carboxyvinyl polymers, which shows a pH around 3. After adding proline, pH increases and the carboxy groups become carboxy ions, which increases electrostatic repulsion and viscosity. Generally, it has been reported that the gelation is caused by the carboxy groups becoming carboxy ions due to the increase in pH [25]. In the case of proline gels, which show a pH around 5, the molar concentration of proline is much higher than that of the carboxy groups of carboxyvinyl polymers, which are sufficiently converted to carboxy ions. Since the proline gels are physically crosslinked gels, the crosslink density depends on the polymer concentration [26]. In this study, the concentration of carboxyvinyl polymers was selected at 0.50 wt%, which is the maximum concentration due to the low solubility of carboxyvinyl polymers in water. Although it is possible to produce proline gels at lower concentrations of carboxyvinyl polymers, the proline concentration should be increased to over 500 μmol g^−1^. However, for the application of tissue regeneration, cytotoxicity by high osmotic pressure should be considered (see Section 2.4). Therefore, the polymer concentration (crosslinked density) was adjusted to 0.50 wt% in the following experiments.

### 2.2. Degradation and Release Behavior of Proline Gels

**Figure 2:**
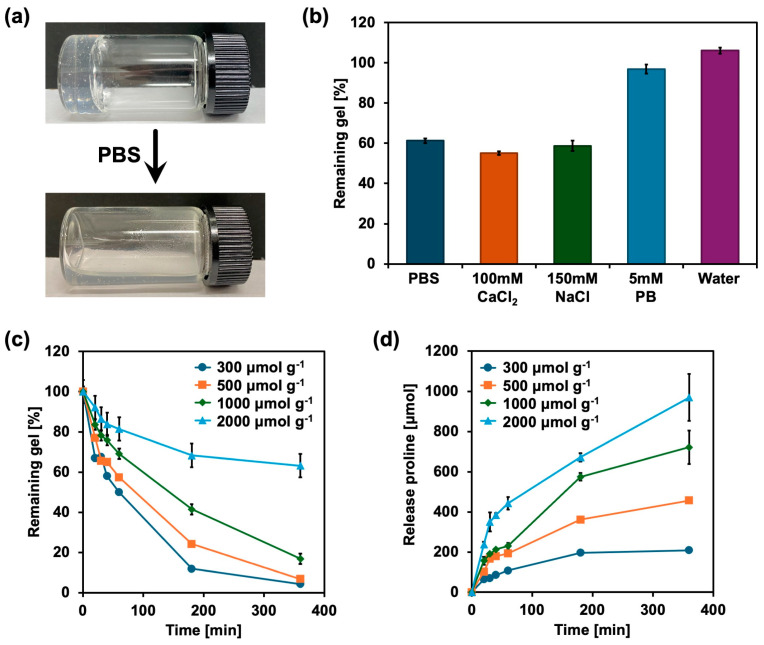
Degradation and release behavior of proline gels. (**a**) Degradation of 500 μmol g^−1^ proline gel after adding PBS. (**b**) Proportion of 500 μmol g^−1^ proline gel remaining after contact with PBS, 100 mM CaCl_2_, 150 mM NaCl, 5 mM phosphate buffer (PB), and water for 1 h (*n* = 3). (**c**) Degradation of 300, 500, 1000, and 2000 μmol g^−1^ proline gels after adding PBS (*n* = 3). (**d**) Amount of proline released from 300, 500, 1000, and 2000 μmol g^−1^ proline gels after adding PBS (*n* = 3).

The degradation of proline gels in serological environments is important for stomatitis healing. As shown in Figure 2a, the 500 μmol g^−1^ proline gel readily degraded upon contact with phosphate buffered saline (PBS, pH 7.4), and all proline gels similarly degraded after contact with PBS. Various solutions were added to the 500 μmol g^−1^ proline gel to evaluate the effect of pH and other salts on the degradation of proline gels. As shown in Figure 2b, the proline gel similarly degraded upon contact with 100 mM CaCl_2_ solution and 150 mM NaCl solution. However, the proline gel did not degrade upon contact with water and 5 mM phosphate buffer (PB), which had the same pH as PBS. This indicates that the proline gel is not degraded by the effect of low osmotic pressure or pH, but rather is degraded by the effect of serological high salt concentrations. FTIR spectra (Figure 1b) suggest that ionization of the carboxy group of carboxyvinyl polymer is the driving force of gelation, and when the gel comes into contact with PBS, which contains abundant ions, the solubility of carboxyvinyl polymer increases to the extent that it cannot retain water inside its polymer network.

Figure 2c shows the degradation profiles of 300, 500, 1000, and 2000 μmol g^−1^ proline gels in PBS. All proline gels gradually degraded within 360 min. After 360 min, almost all 300 and 500 μmol g^−1^ proline gels were dissolved in PBS, and approximately 80% of the 1000 μmol g^−1^ proline gel and 40% of the 2000 μmol g^−1^ proline gel were degraded. The release of proline from the gel phase was determined using an amino acid analysis system. Figure 2d shows the release profiles of proline from the gels. Although the degradation rates of the 300 and 500 μmol g^−1^ proline gels were high, the cumulative amount of proline released from these gels was lower than that from the 1000 and 2000 μmol g^−1^ proline gels. Therefore, the release of proline depends on the concentration of proline in the gel, and proline release was not enhanced by gel degradation. It is known that diffusion is involved in the release of substances from degrading gels [27,28]. The release profiles were fitted to a semi-empirical equation of the Korsmeyer–Peppas model to determine the reaction rate constant *k* and release exponent *n* (Table 2). The fitting results showed that the release exponent *n* ranged from 0.46 to 0.60 for proline gels with proline concentrations of 300 μmol g^−1^ to 2000 μmol g^−1^. According to previous studies [27,28], when the release exponent *n* is 0.45, the reaction is dominated by Fick’s diffusion and when *n* is 0.45 < *n* < 1.00, the reaction is dominated by non-Fickian diffusion, whereas when *n* = 1.00, the reaction follows zero-order kinetics. Therefore, the release of proline from all proline gels is dominated by non-Fickian diffusion. Moreover, the reaction rate constant *k* for proline gels decreased as the proline concentration increased. The 300 μmol g^−1^ proline gel, which has the highest decomposition rate, has the largest reaction rate constant *k*, which is consistent with the degradation results.

### 2.3. Stomatitis Healing In Vivo

**Figure 3:**
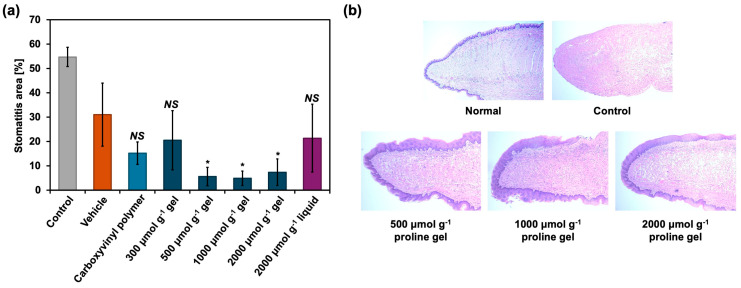
Stomatitis healing in vivo. (**a**) Reduction in the stomatitis area prepared by administering 5-FU and acetic acid on the tongue, as evaluated with toluidine blue staining. Mice without treatment (Control) (*n* = 4) and mice treated with the vehicle (*n* = 4), 0.50 wt% carboxyvinyl polymer (*n* = 4), 300, 500, 1000, and 2000 μmol g^−1^ proline gels (*n* = 4), and 2000 μmol g^−1^ proline aqueous solution (liquid) (*n* = 7) were compared. The stomatitis area was measured 4 days after the administration of acetic acid. * *p* < 0.05 and NS (not significant) vs. vehicle. (**b**) HE stained cross-sections of tips of tongues with stomatitis with or without treatment. Normal: without treatment or administration of 5-FU and acetic acid.

Control samples and 300, 500, 1000, and 2000 μmol g^−1^ proline gels were applied to the tongues of mice. Moreover, 5-FU was administered by intraperitoneal injection, and acetic acid was topically applied on their tongue, establishing stomatitis model mice. The applied samples were (1) a vehicle containing an antibacterial agent (ethyl parahydroxybenzoate aqueous solution), (2) 0.50 wt% carboxyvinyl polymer aqueous solution, (3) 300, 500, 1000, and 2000 μmol g^−1^ proline gels, and (4) 2000 μmol g^−1^ proline liquid (aqueous solution). Figure 3a shows the measured stomatitis area on the tongue after 4 days from treatment with acetic acid, which was determined from the ulcer area stained with toluidine blue. None of 0.50 wt% carboxyvinyl polymer aqueous solution, 300 μmol g^−1^ proline gel, or 2000 μmol g^−1^ proline aqueous solution outperformed the vehicle in reducing the stomatitis area. However, 500, 1000, and 2000 μmol g^−1^ proline gels reduced the stomatitis area by a greater amount than the vehicle. These results indicate that proline gels with sufficiently high concentrations of proline are effective in stomatitis healing. In the case of the 300 μmol g^−1^ proline gel and 2000 μmol g^−1^ proline aqueous solution, proline and water could not stay in the stomatitis area owing to their low viscosity, decreasing the effect of proline or hydration on stomatitis healing. Under the same experimental condition, the stomatitis area was reduced by the same amount following treatment with 500, 1000, and 2000 μmol g^−1^ proline gels, indicating that the amount of proline released from the 500 μmol g^−1^ proline gel is enough to achieve a therapeutic effect, and the excess amount of proline molecules released from the 1000 or 2000 μmol g^−1^ proline gel neither significantly inhibits nor enhances the therapeutic effect.

Histological analysis following hematoxylin–eosin (HE) staining showed that, in the cross-section of the tip of tongue without treatment, 5-FU administration, or acetic acid application (Normal), the layer of mucosal epithelium was present on the outermost surface. However, in the Control sample, in which stomatitis was established by administering 5-FU and acetic acid, the layer of mucosal epithelium disappeared from the outermost surface. In cross-sections treated with 500, 1000, and 2000 μmol g^−1^ proline gels, the mucosal epithelium was present on the tip of tongue, indicating that the mucosa was regenerated by the proline gels.

### 2.4. Cytotoxicity and Cell Proliferation In Vitro

**Figure 4:**
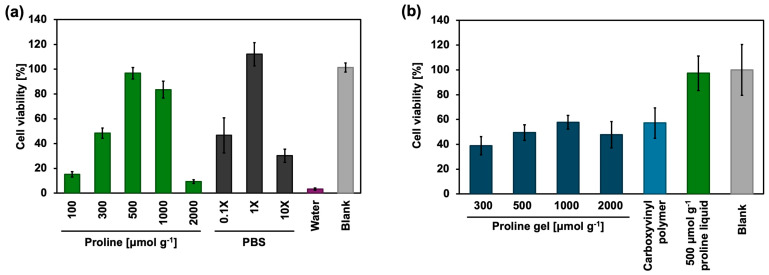
Viability and proliferation of HaCaT cells in vitro. (**a**) Viability of HaCaT cells after 2 h of incubation with 100, 300, 500, 1000, and 2000 μmol g^−1^ proline aqueous solutions, 0.1×, 1×, and 10× PBS, and water (*n* = 3). (**b**) Viability of HaCaT cells after 1 day of incubation with 300, 500, 1000, and 2000 μmol g^−1^ proline gels, 0.50 wt% carboxyvinyl polymer aqueous solution, and 500 μmol g^−1^ proline aqueous solution (*n* = 3).

The cytotoxicity of proline aqueous solutions (100, 300, 500, 1000, and 2000 μmol g^−1^), PBS (0.1×, 1×, and 10×), and water was evaluated using HaCaT cells, as shown in Figure 4a. Among the proline aqueous solutions, the 500 μmol g^−1^ proline solution exhibited the lowest cytotoxicity and the viability of cells exposed to 500 and 1000 μmol g^−1^ proline aqueous solutions was over 80%, indicating that these two solutions were not toxic to HaCaT cells. The main factor determining the toxicity of proline aqueous solutions to HaCaT cells seems to be the osmotic pressure. To determine the effect of osmotic pressure on cells, the viability of cells exposed to PBS (0.1×, 1×, and 10× concentrations) and water was evaluated. The result indicated that 1× PBS maintained the viability of cells at nearly 100%, whereas 0.1× and 10× PBS and even water exhibited cytotoxicity to HaCaT cells, and this phenomenon likely occurred with the proline aqueous solution as well.

The proliferation of cells incubated with 300, 500, 1000, and 2000 μmol g^−1^ proline gels, 0.50 wt% carboxyvinyl polymer aqueous solution, and 500 μmol g^−1^ proline aqueous solution was examined (Figure 4b). After 1 day, proline gels and 0.50 wt% carboxyvinyl polymer aqueous solution had the same effect on cell viability. In contrast, 500 μmol g^−1^ proline aqueous solution maintained the viability of cells at almost 100%. These results indicate that proline is not cytotoxic but carboxyvinyl polymer is, and the inhibition effect of proline gels on cell growth is small.

The above experiments on cytotoxicity, cell proliferation, and in vivo stomatitis healing provided consistent results, namely that 500 and 1000 μmol g^−1^ proline aqueous solutions were less cytotoxic and reduced the stomatitis area by a greater extent than the other samples. Interestingly, the 2000 μmol g^−1^ proline aqueous solution was highly cytotoxic in vitro, and its gel counterpart significantly reduced the stomatitis area in vivo. This confirms that factors such as providing a long-term moist environment surrounding stomatitis are as important as cytotoxicity in stomatitis healing.

## 3. Conclusions

Proline gels consisting of proline, carboxyvinyl polymer, and water were prepared and characterized using the qualitative tilting method and quantitative FTIR, pH, viscosity, and viscoelasticity measurements. The results indicated that proline gels with proline concentrations over 300 μmol g^−1^ formed hydrogels that retained water and hydrated the tongue in the stomatitis model. Moreover, proline gels did not degrade under the action of low osmotic pressure or neutral pH but degraded in response to serologically high salt concentrations, suggesting that ionization of the carboxy group of carboxyvinyl polymer is the driving force of gelation. Additionally, the release of proline from proline gels was governed by the proline concentration, and proline release was not enhanced by gel degradation. In the anticancer drug-induced stomatitis model in vivo, proline gels with proline concentrations from 500 to 2000 μmol g^−1^ provided a therapeutic effect. The combined results of experiments on cytotoxicity, cell proliferation in vitro, and stomatitis healing in vivo suggest that although cytotoxicity is one parameter affecting stomatitis healing, other parameters, such as long-term wetting, may play a role in future developments in stomatitis therapy under anticancer administration.

## 4. Materials and Methods

### 4.1. Preparation of Proline Gels

L-proline and carboxyvinyl polymer (Hiviswako 103) were purchased from Fujifilm Wako Pure Chemicals (Osaka, Japan). Proline was added to water to prepare proline aqueous solutions with desired concentrations. Carboxyvinyl polymer was dispersed in water and stirred at 75 °C for 15 min to prepare 1.0 wt% carboxyvinyl polymer aqueous solution. Each of the proline aqueous solutions and the carboxyvinyl polymer aqueous solution were mixed in a 1:1 weight ratio to produce proline gels with different proline concentrations and 0.50 wt% carboxyvinyl polymer. To determine the qualitative strength of the proline gels, 25 and 30 g of weight per 4.2 cm^2^ (contact area) were placed on 500 and 2000 μmol g^−1^ proline gels.

### 4.2. FTIR and Raman Spectrum

Proline aqueous solution, carboxyvinyl polymer aqueous solution, and proline gel were prepared and freeze-dried (FDU-1200, EYELA, Bohemia, NY, USA). FTIR spectra were acquired using a spectrometer equipped with a diamond attenuated total reflection accessory (FT-720, HORIBA, Kyoto, Japan). The solid powders and aqueous solution of proline, carboxyvinyl polymers, and proline gels were set on the slide glasses, and Raman spectra were measured (LabRAM HR800, HORIBA, Kyoto, Japan).

### 4.3. Gelation, pH, Viscosity, and Viscoelasticity Measurements of Proline Gels

Proline gels with proline concentrations of 10, 50, 100, 200, 300, 400, 500, 1000, 1500, 2000, and 2500 μmol g^−1^ were prepared, and gelation was checked using the tilting method. Briefly, 1.0 mL of proline gel was placed in a No. 2 microtube (Maruemu Corporation, Osaka, Japan). The pH of the proline gels was measured using a pH meter (LAQUA F-71, HORIBA, Kyoto, Japan) and pH electrode (LAQUA 9615S, HORIBA, Kyoto, Japan). The viscosity of the proline gels was measured at 37 °C using a viscometer (TV-200EH, Toki Sangyo, Tokyo, Japan) and 3° × R14 cone rotor at 10 rpm. Viscoelastic measurements were conducted using a dynamic shear rheometer (MCR302, Anton Paar, Graz, Austria) with 8 mm diameter stainless steel parallel plates at 37 °C.

### 4.4. Degradation and Release Behavior of Proline Gels

First, 3.0 g of 500 μmol g^−1^ proline gel was placed in a 15 mL vial and centrifuged at 1000 rpm for 4 min. Then, 0.30 g of 1XPBS was placed on the proline gel. Degradation of the proline gel was observed after 1 h. 100 mM CaCl_2_ aqueous solution, 150 mM NaCl aqueous solution, and 5 mM PB solution were prepared. Then, 1.0 g of 500 μmol g^−1^ proline gel was placed in a 2.0 mL tube (132-620C, WATSON Bio Lab, San Diego, CA, USA), and, after centrifugation, 1.0 g of each of prepared solution, namely CaCl_2_, NaCl, PB, PBS, and water, was placed on the proline gel for 1 h of incubation at 37 °C. To analyze time-dependent degradation by PBS, the remaining gel was calculated using Equation (1). After *t* min, the liquid on top of the proline gel was removed and weighed, denoted as weight of solution (*t* min) in Equation (1).

Remaining gel at *t* min [%] = ((weight of gels at 0 min) − weight of solution (*t* min)) × 100 (1)

The concentration of proline released from proline gels was determined using an amino acid analysis system (NA model, Shimadzu, Kyoto, Japan) equipped with an in-line fluorescent detector (ex. 350 nm, em. 450 nm) and column (Shim-pack Amino Na), with amino acid mobile phase kit and OPA reagent kit. The total flow was 0.8 mL min^−1^. The kinetics were analyzed according to the Korsmeyer–Peppas model, as expressed by Equation (2). *M_t_* and *M_∞_* are the amount of proline released after *t* min of incubation and the maximum amount of proline released from each proline gel, respectively.

(2) $$\frac{M_{t}}{M_{\infty }}=kt^{n}$$

### 4.5. Stomatitis Healing In Vivo

Stomatitis on the tongue of mice (ICR, 9-week-old, female, purchased from CLEA Japan Inc., Tokyo, Japan) was established by administering 5-FU and acetic acid as follows: 5-FU was intraperitoneally injected on days −2, 0, and +2, and 50% acetic acid aqueous solution was applied to the tongues of mice on day 0. The following samples were applied to the tongues of mice twice a day from days −2 to +4: 300, 500, 1000, and 2000 μmol g^−1^ proline gels, vehicle containing antibacterial agent ethyl parahydroxybenzoate aqueous solution at saturated concentration (Fujifilm Wako Pure Chemicals, Osaka, Japan), 0.50 wt% carboxyvinyl polymer aqueous solution, and 2000 μmol g^−1^ proline aqueous solution (liquid). On day +4, the tongues of the mice were harvested, and 1% toluidine blue in acetic acid aqueous solution was added. The percentage of blue colored stomatitis area was calculated using ImageJ (version 1.53t, released from National Institutes of Health, MD, USA). Cross-sections of tongues were stained with HE using standard protocols.

### 4.6. Cytotoxicity and Cell Proliferation In Vitro

Immortalized human keratinocyte (HaCaT) cell lines (Cell Bank, RIKEN BioResource Center, Tsukuba, Japan) in D-MEM/Ham’s F-12 medium (Fujifilm Wako Pure Chemicals, Osaka, Japan) containing 10% fetal bovine serum (CORNING, Corning, NY, USA) and a standard antibiotic solution of penicillin, streptomycin and amphotericin B (Nacalai Tesque, Kyoto, Japan) were placed in cell flasks and incubated at 37 °C in a 5% CO_2_ incubator. For cytotoxicity experiments, HaCaT cells were seeded in 96-well culture plates at 6.0 × 10^3^ cells/well, and after incubation overnight, the medium was collected and incubated with 100, 300, 500, 1000, and 2000 μmol g^−1^ proline aqueous solution, 0.1×, 1×, and 10× PBS, and water. After 2 h of incubation, the samples were collected, and a viable cell counting reagent (Cell Count Reagent SF, Nacalai Tesque, Kyoto, Japan) was added. After 2 h of incubation, the absorbance at 450 nm was measured using a microplate reader (Multiskan FC, Thermo Fisher Scientific, Waltham, MA, USA) to determine the percentage of viable cells. For cell proliferation experiments, HaCaT cells were seeded in 96-well culture plates at 5.0 × 10^3^ cells/well, and after incubation overnight, the medium was collected and incubated with 300, 500, 1000, and 2000 μmol g^−1^ proline gels, 0.50 wt% carboxyvinyl polymer aqueous solution, and 500 μmol g^−1^ proline aqueous solution, which were diluted 10-fold with D-MEM/Ham’s F-12 medium.

### 4.7. Statistical Analysis

Data are shown as average ± standard deviation. Statistical analyses, namely one-way analysis of variance (ANOVA) with Tukey’s post-hoc test, were performed using SPSS version 29.0 (IBM, Armonk, NY, USA).

## Figures and Tables

**Table 1:** Sol–gel states of mixtures of 0.50 wt% carboxyvinyl polymer and various concentrations of proline determined using the tube tilting method.

Proline [μmol g^−1^]	10	50	100	200	300	400	500	1000	1500	2000	2500
State	Sol	Sol	Sol	Sol	Gel	Gel	Gel	Gel	Gel	Gel	Gel

**Table 2:** Release parameters calculated using the semi-empirical equation of the Korsmeyer–Peppas model (*n* = 3).

Proline Gels	*n* [-]	*k* [min^−*n*^]
300 μmol g^−1^	0.46 ± 0.01	0.059 ± 0.007
500 μmol g^−1^	0.51 ± 0.16	0.041 ± 0.021
1000 μmol g^−1^	0.58 ± 0.09	0.025 ± 0.005
2000 μmol g^−1^	0.60 ± 0.02	0.014 ± 0.000

## Data Availability

Dataset available on reasonable request from the corresponding authors.
